# Silencing Abnormal Wing Disc Gene of the Asian Citrus Psyllid, *Diaphorina citri* Disrupts Adult Wing Development and Increases Nymph Mortality

**DOI:** 10.1371/journal.pone.0065392

**Published:** 2013-05-29

**Authors:** Ibrahim El-Shesheny, Subhas Hajeri, Ibrahim El-Hawary, Siddarame Gowda, Nabil Killiny

**Affiliations:** 1 Department of Entomology and Nematology, Citrus Research and Education Center, IFAS, University of Florida, Lake Alfred, Florida, United States of America; 2 Department of Plant Pathology, Citrus Research and Education Center, IFAS, University of Florida, Lake Alfred, Florida, United States of America; 3 Department of Plant Protection, Faculty of Agriculture, Tanta University, Tanta, Egypt; United States Department of Agriculture, United States of America

## Abstract

Huanglongbing (HLB) causes considerable economic losses to citrus industries worldwide. Its management depends on controlling of the Asian citrus Psyllid (ACP), the vector of the bacterium, *Candidatus* Liberibacter asiaticus (*C*Las), the causal agent of HLB. Silencing genes by RNA interference (RNAi) is a promising tool to explore gene functions as well as control pests. In the current study, abnormal wing disc (*awd*) gene associated with wing development in insects is used to interfere with the flight of psyllids. Our study showed that transcription of *awd* is development-dependent and the highest level was found in the last instar (5^th^) of the nymphal stage. Micro-application (topical application) of dsRNA to 5^th^ instar of nymphs caused significant nymphal mortality and adult wing-malformation. These adverse effects in ACP were positively correlated with the amounts of dsRNA used. A qRT-PCR analysis confirmed the dsRNA-mediated transcriptional down-regulation of the *awd* gene. Significant down-regulation was required to induce a wing-malformed phenotype. No effect was found when dsRNA-*gfp* was used, indicating the specific effect of dsRNA*-awd*. Our findings suggest a role for *awd* in ACP wing development and metamorphosis. *awd* could serve as a potential target for insect management either via direct application of dsRNA or by producing transgenic plants expressing dsRNA*-awd*. These strategies will help to mitigate HLB by controlling ACP.

## Introduction

Flying ability is an important evolutionary transition. It requires combination of morphological, physiological, and behavioral features [1]. Only four groups of animals have developed the ability to fly. The three extant groups are bats, birds, and insects. The latter two groups have true wings [2]. However, insects are the “only group of invertebrates that includes members capable of active flight” [3].

Many proteins and hormones and their analogues are implicated in the development of wings and flying ability [4], [5], [6], [7], [8], [9], [10], [11], [12], [13]. Wing discs and other imaginal discs, are cells in the larval stage of insects that remain diploid and continue to divide by normal division in order to differentiate into adult organs [6]. The abnormal wing disc (*awd*) gene of *Drosophila* encodes a soluble protein with nucleoside diphosphate kinase (NDPK) activity. The main function of NDPK is to catalyze the exchange of phosphate groups between different nucleoside diphosphates [14]. The *awd* also regulates wing development in insects such as *Drosophila melanogaster*, *Bombyx mori* and *Antheraea pernyi* [6], [15], [16]. Complete loss of AWD/NDPK function (null mutation) in *D. melanogaster* causes lethality after the larval stage. Most larval organs in this null mutant appear to be normal, but the imaginal discs are small and incapable of normal differentiation [17], [18]. The product of *awd* in *Antheraea pernyi* has also been shown to contribute to insect temperature tolerance [16].

Silencing genes by RNA interference (RNAi) is a promising tool for controlling pests [19]. The use of anti-sense (nonsense) RNA strand transcription to inhibit gene activity has been used since the 80’s [20]. The efficacy of anti-sense silencing depends on hybridization between the injected RNA and endogenous messenger. It was first demonstrated in the nematode *Caenorhabditis elegans* [21] where the double-stranded RNA (dsRNA) produced interference more effectively than individual strands [22]. Subsequently, RNAi using dsRNA was attempted in insect research, but initially it was difficult to achieve silencing. For example, the percentages of abnormal larvae of *Spodoptera exigua* were 11.67% and 31.67% after 24 hours and 72 hours post- injection with dsRNA of chitin synthase gene A, respectively [23], [24].

Many RNAi studies in insects relate to insect immunity [25], [26], [27], embryonic development [28], [29], [30], metamorphosis [9], [31], [32], integument and morphogenesis [33], [34], [35], [36], and communication and behavior [37], [38], [39], [40].

RNAi experiments have been conducted on insects that belong to different taxonomic groups, particularly Holometabolous insects: Lepidopterans; *Bombyx mori*, *Manduca sexta*, *Helicoverpa armigera* [41], *Spodoptera Exigua* [23], [42] *Plutella xylostella* [26], dipterans; *D. melanogaster*, *Bactrocera Dorsalis* [43], and coleopterans; *Diabrotica virgifera* [44], [45]. Fewer studies have been carried out on Hemimetabolous insects: Isoptera; termite *Reticulitermes flavipes* [46], Orthoptera; *Locusta migratoria* [47] *Gryllus bimaculatus* [40]; Hemiptera and Homoptera; *Rhodnius prolixus* [48], *Nilaparvata lugens* [49], [50], *Laodelphax striatellus* [50], *Bemisia tabaci* [51] *Bacterocera cockerelli* [52] and *Acyrthosiphon pisum* [53]. RNAi using dsRNA has been shown to be innovative as a potential alternative technique in insect management compared to insecticide application.

Huanglongbing (HLB), or citrus greening, seriously threatens the citrus industry in Asia, Africa, and the Americas [54], [55], [56], [57], [58], [59], [60]. The First recorded incidence of HLB in Florida was in 2005; since then it has spread rapidly throughout the state [61]. HLB management critically depends on the control of the Asian citrus psyllid (ACP), *Diaphorina citri* Kuwayama (Hemiptera: Psyllidae), the vector of *Candidatus* Liberibacter asiaticus bacteria (*C*Las), the causal agent of HLB. The psyllids are a group of small, plant phloem sap-sucking, hemiptran insects belong to superfamily Psylloidea and family Psyllidae. Along with aphids, scale insects, mealy-bugs, and whiteflies, they belong to the group Sternorrhyncha. There are over 3000 described species of psyllids, and the genus *Diaphorina* contains more than 50 species. *D. citri* is the most serious pest of citrus. Its direct damage occurs from the phloem sap feeding of nymphs and adults, but the real threat is as a vector of *C*las, the causal bacteria of HLB [56], [62]. The psyllid acquires *C*Las bacteria while feeding on infected trees, and then transmits the bacteria after flying to healthy trees for further feeding. Disruption of its flying ability potentially would prevent the spread of HLB. *awd* has been sequenced and annotated for ACP (accession number ABG81980.1). In this study, we report 1) the role of *awd* in ACP development and wing formation and 2) interference of *awd* transcription by micro-application of dsRNA to ACP nymphs.

## Materials and Methods

### Insect culture

Colonies of ACP have been maintained in cages on the sweet orange ‘Valencia’, in temperature controlled growth rooms set at 25±2°C temperature, 60±5% RH, and a 16∶8 (L∶D) photoperiod [63]. For the bioassay and the RT-PCR analysis, seven ACP stages were used and they included 1^st^, 2^nd^, 3^rd^, 4^th^, and 5^th^ nymph instars, and both teneral and mature adults. Different nymph instars were collected in Petri dishes using a hair brush; each nymph was examined morphologically under a stereomicroscope, then classified based on morphological features into its instars [64], [65], [66], [67]. Teneral and mature adults were collected using an aspirator.

### Genetic manipulation

#### In silico analysis of awd

The protein sequence ABG81980.1 from ACP identified as a putative abnormal wing disc-like protein was used to generate a protein sequence multiple alignments with orthologs using Clustal W [68] and BOXSHADE 3.21 (http://www.ch.embnet.org/index.html) to visualize conserved regions in the alignment. The GENO3D server [69] was used to predict secondary structure agreement to validate template selection and alignment by generating a three-dimensional structure and a model for the molecular surface of putative abnormal wing disc-like protein. The 3-D protein-structure model was generated based on identification of 76% (117/152) and positives 90% (137/152) with PDB 1nd1A which represent the structure of the AWD nucleoside diphosphate kinase from *D. melanogaster* in the protein data bank (PDB, http://www.rcsb.org). The predicted macromolecule in the Protein Database File (PDB) was visualized using the software FirstGlance in Jmol (http://molvis.sdsc.edu/fgij/). The prediction of RNA secondary structure for *awd* was performed from the RNA sequence using CentroidFold sequence (http://www.ncrna.org/centroidfold).

#### Gene expression analysis

RNA from nymphal instars and adults was extracted using TriZol reagent and 25 nymphs or adults for each developmental stage or instar with five biological replicates for each. Single stranded RNA was purified using ssDNA/RNA Clean & Concentrator™ (Zymo Research). The concentration and purity of isolated single stranded RNA were determined using NanoDrop. SYBR Green I based quantitative RT-PCR (qRT-PCR) was used to monitor the expression levels of *awd* during different developmental stages of ACP, and also to determine the down-regulation of *awd* in dsRNA treated ACP. Samples were run in triplicate of each biological replicate. PCR primers were, *awd*-RT-F 5′ AGAGGACTTGTGGGAAACATC 3′ and *awd*-RT-R 5′

TGACAAGACCAGGGAAGAAAG 3′. Amplification was performed with a Fast ABI 7500 real-time PCR system (Applied Biosystems) using SYBR green technology. *Alpha-tubulin* was used as a non-target gene (control). To compare the relative gene expression among treatments, we normalized gene expression to *Actin* (reference gene).

#### Synthesizing of the dsRNA

Putative abnormal wing disc-like protein (*awd*) mRNA of ACP is 462 bp in length (GenBank: DQ673407.1). Specific primers (forward primer with PacI; 5′ GCCGAACCCAAGGAAAGAACTTTTCTCATG 3′ and Reverse primer with StuI; 5′ TTATTCATAGATCCAGGATTCACTGGCATTTG

3′ were designed to fully amplify the *awd* gene excluding the start codon. Total RNA from ACP was extracted using Trizol reagent (Life Technologies corp.) as per manufacturer instructions. SuperScript® III One-Step RT-PCR System with Platinum® Taq DNA Polymerase (Life Technologies corp.) was used to amplify the product of 459 bp, that was gel purified using GENECLEAN® III kit (MP Biomedicals) and used for TA cloning into pGEM-T vector (Promega corp.). *awd*/pGEM-T vector was sequenced using universal T7 promoter primer to confirm the cloned AWD sequence. *awd*/pGEM-T vector was linearized by restriction digesting with *Nco*I or *Spe*I to generate sense and antisense transcripts using Ambion® MAXIscript® T7/SP6 In Vitro Transcription Kit (Life Technologies corp.) respectively. To produce *awd* double- stranded (ds) RNAs, sense and antisense transcript were annealed in a single tube by denaturing at 70°C for 10 min followed by slow cooling to room temperature for 20 min. To eliminate the DNA template and single-stranded RNAs, annealed dsRNA was treated with RQ DNase I (Promega corp.) and RNase A (Sigma-Aldrich Co.). The resulting dsRNA was extracted with Phenol:Chloroform:Isoamyl Alcohol (25:24:1, v/v). The amount of purified dsRNA was measured by the NanoDrop spectrophotometer. We used dsRNA-*gfp* as an irrelevant dsRNA (control). dsRNA-*gfp* was produced as described above. Green fluorescent protein (GFP) mRNA is 732 bp in length. Specific primers (Forward primer with SpeI: 5′- GCGA*ACTAGT*ATGGCTAGCAAAGGAGAAGAACTTTTCACTG -3′ and Reverse primer with SacII: 5′- GAGA*CCGCGG*CTACCCCTCGAGTTATTTGTAGAGCTCATC -3′) were used to amplify full-length GFP gene by using TMV-30BGFP.

### Insect bioassay

For all insect bioassays, greenhouse maintained *C*Las-free ‘Valencia’ orange seedlings were used. During the insect assays, ACP adults and nymphs were reared on citrus seedlings of 6 to 8 true leaves stage covered with plastic cylindrical shaped containers (15 cm diameter and 50 cm high). The bottom opening of this cylinder was slipped over the soil around the seedling, and the upper opening was covered with mesh screen for ventilation.

### The application of dsRNA

For ventral epidermal (cuticular) micro-application of dsRNA, the nymphs were collected, and 0.3 µl of dsRNA was applied to individual 5^th^ instar nymphs with a micro- pipette. The dsRNA was placed ventrally on the thorax region between the three pairs of legs and the drop was allowed to be absorbed for approximately 60 seconds. Four concentrations of dsRNA-*awd*: 0.03, 0.3, 3 and 30 µg/µl, were tested. RNAase free water and dsRNA-*gfp* were used as controls. The nymphs were then transferred onto the citrus seedlings, and covered with the plastic cylindrical shaped containers. Five replicates (12 nymphs each) were used for each treatment.

### Abnormality and survival rates determination

Treated nymphs were observed every 24 h to determine the day of adult eclosion and mortality of nymphs. Newly emerged adults were collected and examined for wing malformation and photographed using a Canon Power Shot S3IS digital camera, Leica M3Z stereomicroscope.

### Statistical analysis

Data were analyzed using Minitab® 16.1.0 software. Analysis of variance (ANOVA) was performed on the mean percentages of nymph mortality and wing-malformation with different treatments, followed by means separation according to the Fisher method. All values in figures represent means with standard deviations. Concentration values were logarithmically transformed prior to regression analysis to determine mean percentages of nymph mortality and adult wing malformation.

## Results

### *In silico* analyses of *awd* in ACP

**Figure 1:**
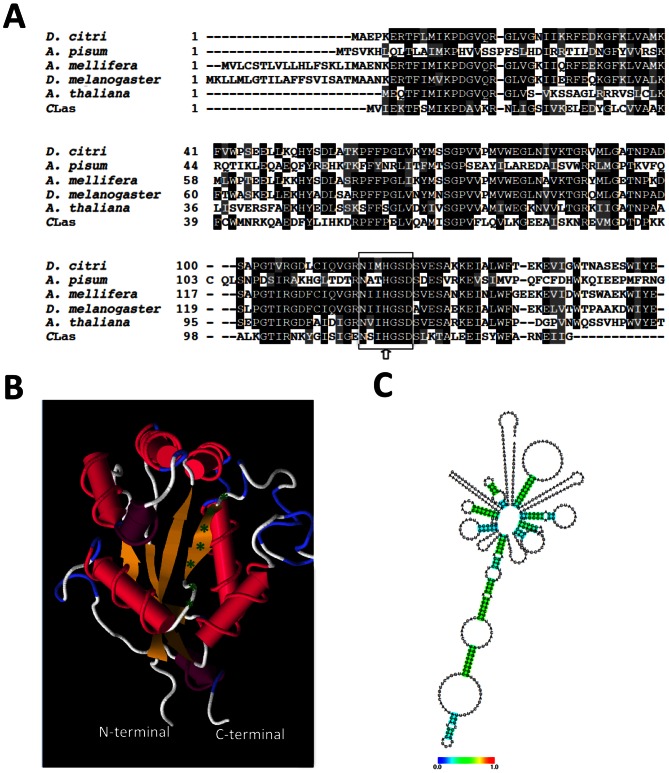
*In silico* analysis of AWD protein sequence and mRNA. Alignment of AWD amino acid sequences from *Diaphorina citri, Acyrthosiphon pisum, Apis mellifera, Drosophila melanogaster, Arabidopsis thaliana, and Candidatus* Liberibacter asiaticus str. psy62. Conserved amino acids are indicated with black shading and those with high similarity score are in gray. The NDPK motif is surrounded by box and pointed to by arrow (A). Predicted three-dimensional structure of AWD with 90% similarity of AWD-NDPK from *Drosophila melanogaster*. NPDK motif is indicated by green asterisks (B). Predicted mRNA hairpin secondary structure of *awd*. Colors represent strengths with base pairing probabilities (C).

The genome of ACP contains *awd* (ABG81980.1) which encodes a putative abnormal wing disc like-protein also known as nucleoside diphosphate kinase (NDPK) containing 153 amino acids with a predicted molecular mass of 17.1 kDa, and 7.82 isoelectric point. The multiple alignment of the amino acid sequence of ACP-AWD protein with the sequences of other known AWD/NPDK revealed that *Apis mellifera,* and *D. melanogaster* showed high similarity and conserved sequences while less similarity was observed with *Acyrthosiphon pisum*. Similarities were also found in plants (*Arabidopsis thaliana*), and CLas str. psy62. Interestingly, we did not find any homology to AWD of ACP in the genome of citrus (*Citrus sinensis* (L.), the main host of ACP. AWD/NDPKs active site motif has been determined to be N-x-x-H-[G/A]-S-D [70], [71]. The motif site amino acid sequence of AWD from ACP was detected in all organisms used for alignment (Fig.1A). The predicted three-dimensional secondary structure of AWD with 90% similarity to AWD/NDPK of *D. melanogaster* and the conserved motif site are shown in Fig. 1B. These results suggest that the motif site of AWD-NDPK is conserved and functional. Predicted mRNA hairpin secondary structure of *awd* and the strengths of base pairing probabilities are shown in Fig. 1C.

### AWD is implicated in ACP Development

**Figure 2:**
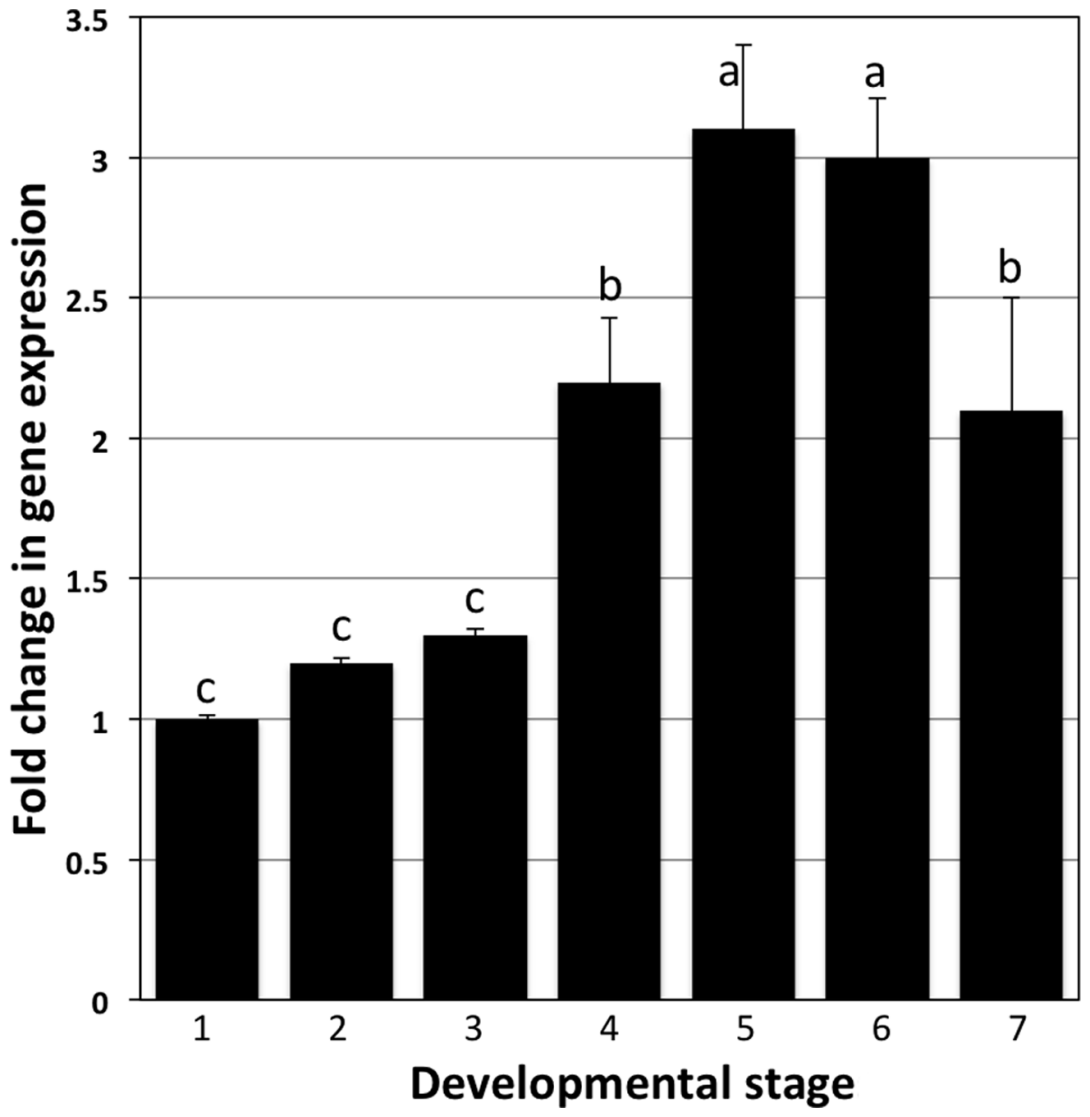
The relative expression level of *awd* using qRT-PCR during different developmental stages of ACP. 1- the 1^st^ instar, 2- the 2^nd^ instar, 3- the 3^rd^ instar, 4- the 4^th^ instar, 5- the 5^th^ instar, 6- the teneral adult, and 7- the mature adult of ACP. Actin gene was used as internal normalization control. Bars represent the standard deviations. Different letters indicate statistically significant differences (*P*<0.05)

Gene expression analysis was carried out to determine the transcription of *awd* at different developmental stages. Stages included 1^st^, 2^nd^, 3^rd^, 4^th^, and 5^th^ nymph instars, and teneral and mature adults. qRT-PCR showed that the expression of *awd* was up-regulated gradually from the 1^st^ nymph instar and reached the highest expression in the 5^th^ nymph instar showing slight down-regulation in the teneral adult stage (Fig. 2). Higher down-regulation was observed in the mature adults. These findings suggest that expression of *awd* varied throughout the nymphal and adult development. The expression of *awd* was observed to be 3 fold higher in 5^th^ instars compared to the 1^st^ instar.

### Silencing of *awd* in nymphs by dsRNA causes increased mortality

**Figure 3:**
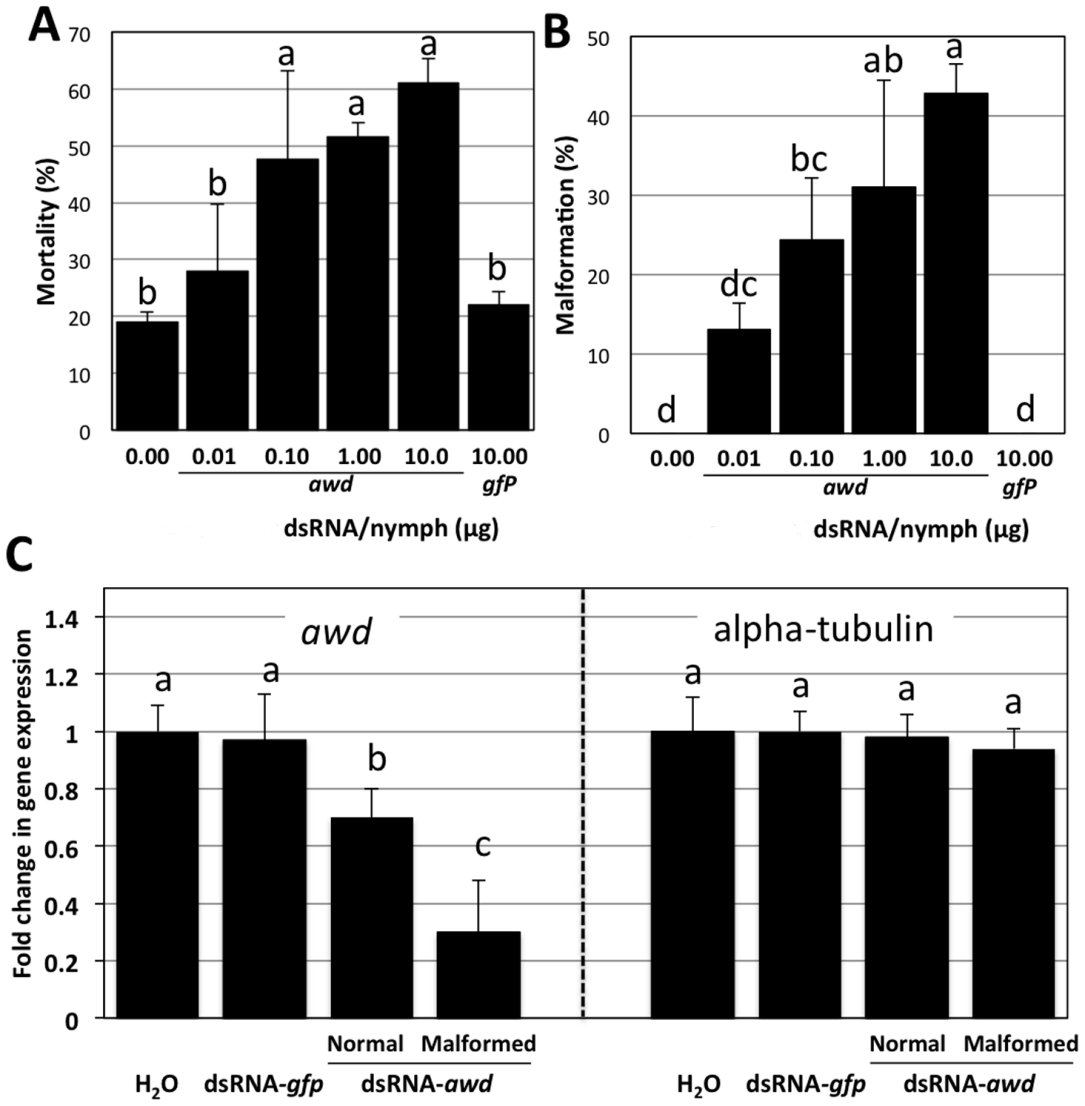
Effects of dsRNA-*awd* on ACP mortality and malformation. Mortality of 5^th^ instar of nymphs treated with different amount of dsRNA (A).Wing-malformed adults developed from treating 5^th^ instars of nymphs with different amount of dsRNA (B). Relative *awd* and *alpha tubulin* expression using qRT-PCR in normal looking and wing-malformed adults obtained from 5^th^ instar nymphs treated with 1μg/nymph of dsRNA-*awd* or 10μg/nymph of dsRNA-*gfp* (C). Each nymph was treated with 0.3ul volume that contains one of the indicated amounts of dsRNA. Bars represent the standard deviations. Different letters indicate statistically significant differences (*P*<0.05)

The main objective of this study is to disrupt wing development in ACP as a means to interfere with psylid flight for prevention of HLB spread in citrus. We targeted *awd* for silencing using dsRNA since it is implicated in the formation of wings in ACP and any interference could inhibit ability to fly. Towards this end, two major biological parameters were investigated: 1) the mortality of nymphs due to the application with dsRNA-*awd*; if the silencing of the *awd* in nymphs would alter their survival and 2) wing malformation in adults. Since the highest expression of *awd* was observed in the 5^th^ instar of nymph, we attempted to silence the expression of *awd* by RNAi in this stage of nymph development. Incremental amounts (ten-folds) of dsRNA-*awd*, 10, 1.0, 0.10, and 0.01 µg, and water as control were applied for each nymph and the experiment was conducted in five replicates. We also used dsRNA-*gfp* as an irrelevant dsRNA (control). dsRNAs was applied via cuticular micro-application, ventrally on thorax region (sternites) between the three pairs of legs of the 5^th^ instar nymphs. There were significant differences in mortality among six treatments (df, 5; F, 7.25; P, 0.004) (Fig. 3A). Significant differences were not observed among the highest three concentrations of dsRNA-*awd* (10, 1.0, 0.10, and 0.01 µg/nymph) which caused mortality of 61±4.58%, 51.52±2.62%, and 47.58±18.77%, respectively. Differences were also not observed between 0.01 µg /nymph of dsRNA-*awd* application and controls (water and dsRNA-*gfp*), showed 27.98±11.68%, 18.89±1.92%, and 23.33±5.77% respectively (Fig. 3A). No significant difference was found between treatment with water and treatment with dsRNA-*gfp* indicating a specific effect of dsRNA-*awd*. A regression coefficient analysis between mortality and log-dose showed a significant positive relationship (b = 10.3, P = 0.042, and R^2^ = 0.92).

### Treatment of ACP nymphs with dsRNA-*awd* causes wing malformations

**Figure 4:**
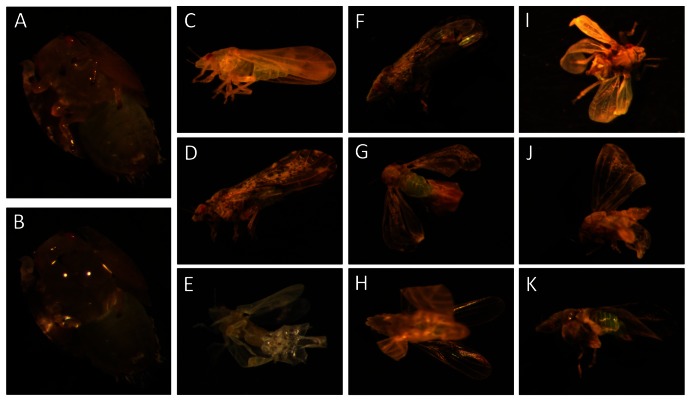
Micro-application of *awd-*dsRNA and its effects on ACP wing formation. The 5^th^ instar nymph on its back side (A). Drop of dsRNA-*awd* solution on the abdominal side between legs in the thorax region of the 5^th^ instar nymph (B), Control ACP teneral adult (C). Control ACP mature adult (D). Different types of wing malformation observed on adults emerged from treated nymphs (E–K). Strongly attached exicuva to teneral adult (E). Malformation in one forewing (F). Both forewings and hindwings are malformed and the adult cannot emerge completely (G). Malformatio in the both forewings but not in hindwings (H). Both forewings and hindwings are malformed (I). All wings malformed and one side is severer than other side. Note also the abnormal dorsal tergites (J). Malformation in one side both forewing and hindwing (K).

Highly significant abnormalities of wing formation in the emerged adults were been observed among treatments (df, 5; F, 11.57; P>0.001) (Fig. 3B). The percentages of wing-malformed emerged adults were 42.68±3.7%, 31±13.42%, 24.41±7.81% and 13.06±3.37% in nymphs treated with concentrations of 30 µg/µl, 3 µg/µl, 0.3 µg/µl and 0.03 µg/µl of dsRNA-*awd* respectively. Thus the application of dsRNA-*awd* affected the development of adult wings. No wing-malformed adults occored in controls (water or dsRNA-*gfp*). The regression coefficient between malformation percentages and Log-dose was significant (b = 9.6, P = 0.001, and R^2^ = 0.99). Various forms of wing malformation were observed (Fig. 4). In some cases, the complete ecolosion of wing-malformed adults was not observed and the exicuvium tightly attached to the teneral adult. Wing malformation sometimes happened in one or both forewings or hind-wings. In some cases, one side or even both sides were malformed. Some abnormalities of dorsal sclerites in thorax tergites were observed (Fig. 4). We also noticed that wing-malformed adults could not move normally or fly. These results suggest an important role for the AWD/NPDK in the development of wings in ACP.

### Wing malformation depends on degree of silencing

To demonstrate silencing of *awd* at the molecular level, analysis of *awd* expression was conducted by qRT-PCR. Three types of adults were examined; normal looking adults developed from treated nymphs; wing-malformed adults developed from treated nymphs and adults from control nymphs. qRT-PCR results demonstrated that *awd* is down-regulated in both types of adults that developed from dsRNA-*awd* treated nymphs compared to the control (Fig. 3C). A pronounced down-regulation of *awd* was found in wing-malformed adults with 70% of reduction of expression level compared to control groups. On the other hand, the expression of *awd* was also down-regulated in normal-looking adults developed from treated nymphs but not to the same level as in the wing-malformed adults. These findings suggest that a significant gene down-regulation is required to induce the malformed wing phenotype.

## Discussion

Determination of the suitable stage for dsRNA treatment is crucial for inducing silencing in ACP. In this study, the 5^th^ instar nymphs were chosen to be the optimal stage for dsRNA-*awd* application for silencing the endogenous psyllid gene (Fig. 4A, C). Silencing has been observed in specific stage and/or specific instars of insects, and not others [48], [72]. This may relate first to the nature of the targeted gene and its functions, then susceptibility of different stages to RNAi and the suitability of delivery method. In the current work, the selected gene, abnormal wing disc gene (*awd)* is known to regulate wing development in insects [6], [15]. Additionally, gene expression at different stages of ACP indicated that the expression of the *awd* gene increased progressively during the nymphal stage until the 5^th^ instars, then decreased in the adults (Fig. 2). This suggests that the product of *awd* is vital during later nymphal stages, and targeting it by RNAi during these stages could potentially affect biological functions. The fact that *awd* expression level was higher during the last instar of the nymphal stage indicates the importance of *awd* in the development and differentiation processes of discs during metamorphosis of ACP. In other insects, *awd* has been implicated in many biological functions such as imaginal discs development, and its complete loss produced lethal mutations and death at the end of the larval stage or during the pupal stage [6], [73], [74].

The delivery of dsRNA is considered to be a limiting factor and must be considered when assessing the effectiveness of RNAi. Silencing potentially would occur once dsRNA is efficiently delivered inside the cell. There are three main methods of dsRNA delivery: injection, ingestion, and epidermal (cuticular) application. In the present study, dsRNA-*awd* was delivered via cuticular micro-application to 5^th^ instars nymph stage. To date, most RNAi studies in insects have been conducted using micro-injection or by feeding through artificial diet systems. Although, micro-injection of dsRNA has been reported, and in some cases silencing occurred successfully as observed in piercing-sucking insects such as Hemiptera and Homoptera, the silencing levels varied widely [24], [50], [75]. In addition, it is hard to deliver dsRNA to the insect hemocoel via micro-injection to small and soft insect stages. Therefore, feeding of dsRNA is a more common approach than micro-injection. But, the success of RNAi through feeding application usually requires high amounts of dsRNA since it is suitable to degradation during feeding and inside the insect gut. Few studies using feeding delivery were carried out with psyllids, and silencing was not successful with some of the target genes [52]. It would be very useful if the silencing signal passes through the insect gut systemically to other tissues. In most cases of successful silencing studies, the mid-gut is the primary target organ and mid-gut tissue genes were the main targets [19], [37], [76]. However, Some systemic silencing after dsRNA delivery through feeding has been observed [40].

Insect cuticle might appear refractory to dsRNA application, since no silencing studies were carried out in insects via cuticular micro-application. In targeting epidermis and disruption of chitin synthesis during molting, micro-injection has been used [23]. However, our study demonstrates that ventral cuticular micro-application of dsRNA-*awd* allowed dsRNA uptake through the exoskeleton of the psyllid nymphal stage. The uptake was probably occurred via inter-segmental membranes especially in ventral portions (sterna), through membranes between articulation plates and cavities of appendage bases (legs and wing) and through the lateral portions on each side (pleura) including the spiracles. We have thus shown that micro-application of dsRNA could be an alternative method for the delivery of dsRNA to soft bodied stages in the soft and tiny insect life cycle. The flexible membranes between sclerites and around articulation cavities especially in soft cuticle of immature insects contain a thin exocuticle which make these parts soft and water-permeable [77], [78], [79], [80]. It was reported that even low polarity substances penetrate the insect cuticle through intersegmental membranes [81], wax and pore canals [82]. The complete morphology and the arrangement of the dorsal and ventral sclerites of ACP 5^th^ instar had been described in detail by White and Honkinson [83]. The ventral side of the 5^th^ nymphal instar of psyllids has less sclerites and more membrane area between plates and around articulation cavities of legs exist than on dorsal side. Indeed, the dorasal side has the prothoracic tergum continuous with the dorsal surface of the mesothoracic. Most of the sclerites are fused with the head to form the ‘cephaloprothorax’. Psyllida have at least 2+2 sclerites between the mesothorax and cephaloprothorax. The mesothorax tergum is completely fused. Metathorax also, has the same form as the mesothorax. The abdominal sclerites are arranged at least 1+1 per segment, but in the epical area the individual sclerites are often fused, whereas, the ventral abdominal surface of most species has 2+2 sclerites and the areas between these sclerites are membranous [83].

The results presented here clearly demonstrate that dsRNA is a powerful agent for gene silencing and mediates post-transcriptional down-regulation of *awd* transcripts as confirmed through qRT-PCR and the morphological changes in some adults developing from treated nymphs. We also measured positive correlation between the dsRNA concentrations and mortality and wing malformation. A relationship between dsRNA concentrations and silencing level probably is determined by the method of dsRNA delivery and the group of insects involved. Whereas, correlations between dosage and effect iveness of RNAi have been shown in some dsRNA injection studies [19], [84], [85], it did not occur in lepidopteran insects, perhaps due to systemic RNAi sensitivity/resistance differences among different groups of insects [24].

The results of gene expression analysis indicated that *awd* is down-regulated in both normal and wing-malformed adults obtained from treated nymphs compared to the control (Fig. 3C). A higher down-regulation of *awd* in wing-malformed adults was found. The malformed wing phenotypes resulting from the RNAi are probably caused by down-regulation/mal-functionality of many important metabolic pathway intermediates, especially those related to wing formation. Down-regulation of *awd* in normal looking adults that developed from treated nymphs suggests that higher levels of silencing are required to affect wing-malformed phenotype.

Further studies are required to improve the micro-application dsRNA delivery and to explore other functions of AWD such as NDPK activity. Pest control strategies using RNAi via dsRNA could be tailored for use against a single pest or groups of related species [19]. We have explored the implication of AWD in wing development, and our results strongly suggest that *awd* could be a potential target for insect management either via direct applicaion of dsRNA or via citrus plants expressing dsRNA against the *awd* gene. RNAi-mediated silencing of some genes of insect pests has been reported in transgenic plants such as transgenic corn expressing V-ATPase A dsRNA against *Diabrotica virgifera* [44] and cotton expressing dsRNA of CYP6AE14 against *Helicoverpa armigera* [86] and these strategies, if applied to citrus, will impact the spread of HLB by controlling the psyllids.
